# The Functional and Molecular Effects of Doxycycline Treatment on *Borrelia burgdorferi* Phenotype

**DOI:** 10.3389/fmicb.2019.00690

**Published:** 2019-04-18

**Authors:** John R. Caskey, Nicole R. Hasenkampf, Dale S. Martin, Vladimir N. Chouljenko, Ramesh Subramanian, Mercedes A. Cheslock, Monica E. Embers

**Affiliations:** ^1^Division of Bacteriology and Parasitology, Tulane National Primate Research Center, Tulane University Health Sciences, Covington, LA, United States; ^2^Division of Biotechnology and Molecular Medicine, School of Veterinary Medicine, Louisiana State University, Baton Rouge, LA, United States

**Keywords:** Lyme disease, antibiotic, *Borrelia (Borreliella) burgdorferi*, RNASeq analysis, mice

## Abstract

Recent studies have shown that *Borrelia burgdorferi* can form antibiotic-tolerant persisters in the presence of microbiostatic drugs such as doxycycline. Precisely how this occurs is yet unknown. Our goal was to examine gene transcription by *B. burgdorferi* following doxycycline treatment in an effort to identify both persister-associated genes and possible targets for antimicrobial intervention. To do so, we performed next-generation RNA sequencing on doxycycline-treated spirochetes and treated spirochetes following regrowth, comparing them to untreated *B. burgdorferi*. A number of genes were perturbed and most of those which were statistically significant were down-regulated in the treated versus the untreated or treated/re-grown. Genes upregulated in the treated *B. burgdorferi* included a number of *Erp* genes and *rplU*, a 50S ribosomal protein. Among those genes associated with post-treatment regrowth were *bba74* (Oms28), *bba03*, several peptide ABC transporters, *ospA, ospB*, *ospC*, *dbpA* and *bba62*. Studies are underway to determine if these same genes are perturbed in *B. burgdorferi* treated with doxycycline in a host environment.

## Introduction

The efficacy of antibiotic treatment for Lyme disease has been a contentious issue among physicians and researchers (DeLong et al., 2012; Halperin, 2015). In particular, the ability of commonly used and recommended therapy regimens to cure disease has been called into question (Cameron et al., 2014). Evidence to indicate that infection is not eradicated by conventional antibiotics such as doxycycline and ceftriaxone has been provided by studies in mice (Hodzic et al., 2008, 2014; Barthold et al., 2010), dogs (Straubinger et al., 1997) and in our own studies of non-human primates (Embers et al., 2012, 2017; Crossland et al., 2018). In actuality, eradication of the spirochetes following antibiotic treatment of a disseminated infection has not been achieved in any animal model tested (Straubinger et al., 1997; Bockenstedt et al., 2002, 2012; Hodzic et al., 2008, 2014; Barthold et al., 2010; Embers et al., 2012). Despite these findings, the mechanisms of post-treatment disease are not well understood (Aucott, 2015). The symptoms which a significant portion of patients experience could result from autoimmunity (Weinstein and Britchkov, 2002), immune responses to residual, dead spirochetes (Bockenstedt et al., 2002, 2012), or a remaining infection. Each of these plausible explanations may also be acting in concert.

Studies on the development of antibiotic-tolerant *Borrelia burgdorferi* persisters have been conducted using *in vitro* experimentation, and research on the development of drug tolerance in general is expanding (Meylan et al., 2018). *B. burgdorferi* has been shown to develop persisters in the presence of doxycycline (Caskey and Embers, 2015), amoxicillin, and ceftriaxone (Sharma et al., 2015). The mechanism by which *B. burgdorferi* persists is driven by stochastically determined slowed growth (Caskey and Embers, 2015) and gene expression is altered (Feng et al., 2015). The ability of this spirochete to enter a slow-growing, dormant phase likely results from the evolution of its zoonotic cycle (Stewart and Rosa, 2018). Within the tick, the spirochetes encounter a nutrient-poor environment which lasts several months. Population growth increases after the blood meal (Piesman et al., 1990), but quickly subsides and becomes stagnant.

Commonly prescribed antibiotics for Lyme disease, including doxycycline and amoxicillin, are also microbiostatic. The mechanism of interfering with protein translation operates upon actively dividing cells, so may be less effective in slow-growing populations. Such antibiotics are able to stop the growth of the bacteria such that the immune system can target and clear the infection. However, *B. burgdorferi* persists in an immune host environment, and the multiple modes of immune evasion (Embers et al., 2004; Hastey et al., 2012) may also reduce the effectiveness of microbiostatic antibiotics.

In the set of experiments described in this report, two keys aspects of doxycycline-treated *B. burgdorferi* phenotype were evaluated. First, the functionality of antibiotic-treated spirochetes was assessed by testing infectivity in immune-deficient and immune-competent murine hosts. Second, the molecular adaptation to doxycycline treatment was comprehensively investigated with next-generation sequencing to obtain transcriptional profiles of spirochetes that were treated and those that were treated and re-grew. These studies allow us to better understand both functional and molecular phenotypes to drug-tolerant *B. burgdorferi* persisters and to identify potential targets for treatment.

## Materials and Methods

### Ethics Statement

Practices in the housing and care of mice conformed to the regulations and standards of the Public Health Service Policy on Humane Care and Use of Laboratory Animals, and the Guide for the Care and Use of Laboratory Animals. The Tulane National Primate Research Center (TNPRC) is fully accredited by the Association for the Assessment and Accreditation of Laboratory Animal Care-International. The Tulane University Institutional Animal Care and Use Committee approved all animal-related protocols, including the infection and sample collection from mice.

#### Borrelia Culture and Antibiotic Treatment Regimen

Low passage (p4 or p5) *Borrelia burgdorferi sensu stricto* strain B31 clonal isolate 5A19 (Purser and Norris, 2000) was grown at 34°C in BSK-II media (Zuckert, 2007), as described previously (Barbour, 1984). Because the spirochetes are microaerophilic and gene expression is affected by oxygen levels (Seshu et al., 2004) they were grown in a tri-gas incubator set at 5% CO_2_, 3% O_2_, and the remainder N_2_. For the infectivity assay in mice, three sets of 50 ml *B. burgdorferi* cultures were inoculated from frozen stocks and grown to 5 × 10^7^ cells/mL. In a previous study, we determined the density-independent minimum inhibitory concentration (MIC) for this strain in a 5 day treatment protocol to be 2.5 μg/ml and the minimum bactericidal dose to be 50 μg/mL (Caskey and Embers, 2015). Thus, the *B. burgdorferi* cultures to be used for the bioassay were either not treated (0 μg/mL), treated with a concentration (10 μg/mL doxycycline) higher than the MIC but not greater than the minimum bactericidal concentration (MBC), or treated with the MBC. In all cases, BSK-II media was determined to be the optimal growth media for the experimental conditions after comparing growth rates of *B. burgdorferi* in BSK-II and BSK-H (data not shown). At day 5, the cultures were checked for motility by dark field microscopy (Johnson, 1989) (for RNA seq experiments) and for viability by BacLight staining (for bioassay in mice, as shown in the Supplementary Material).

For RNA sequencing, duplicate *in vitro* experiments to determine gene expression under antibiotic-treated, and antibiotic-withdrawn conditions were conducted with three groups and two biological replicates of each group performed separately. The spirochetes were grown to 5 × 10^7^ cells/mL as described (Caskey and Embers, 2015). Group 1, (untreated) consisted of RNA from an untreated *B. burgdorferi* control. Group 2 (treated), consisted of RNA from *B. burgdorferi* treated with 50 μg/mL doxycycline for 5 days. Group 3 (treated/re-grown), consisted of RNA from *B. burgdorferi* treated with 50 μg/mL doxycycline for 5 days, then allowed to regrow until the population reached the initial pre-treatment concentration of 5 × 10^7^ cells/ml; this re-growth occurred between 10 and 12 days after the end of the treatment period.

#### Bioassay in Mice

**Figure 1:**
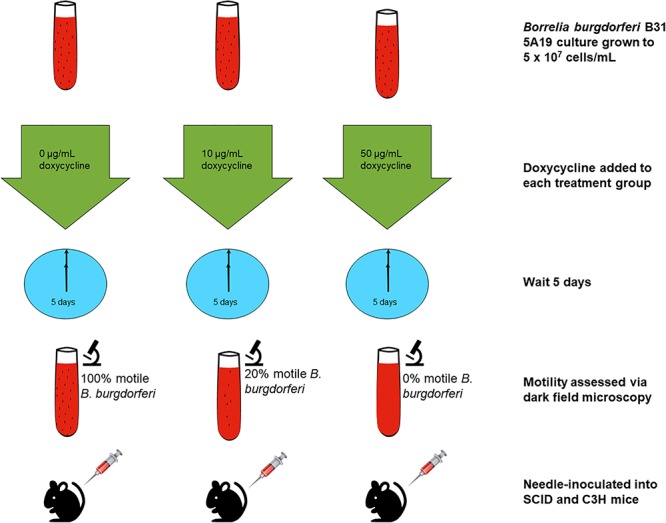
Experimental outline for the assessment of infectivity in mice. *B. burgdorferi* cultures were treated for 5 days with doxycycline or left untreated. The spirochetes were then injected into mice that were later evaluated for infection.

A sample of 2 × 10^5^ *B. burgdorferi* derived from one of the three treatment groups was needle-inoculated into severe combined immune-deficient CB-17. SCID mice and C3H/HeN mice (Charles River Labs). Ear punch biopsies (2 mm) were taken on day 14 for all mice. On day 21, the mice were euthanized, and tissues including ear skin, heart, bladder, spleen and tibiotarsal joints were harvested. Tissues were used for culture in 5 mL of BSK-II and preserved in RNALater^TM^ to perform RT-PCR for both *flaB* and *ospC* genes. This was not performed in one experimental set of mice. The culture tubes were incubated in a tri-gas incubator as described above, and checked 2–3 times weekly, for up to 45 days, for signs of motility. The experiment was repeated three times, with a total of five control group SCID mice and C3H mice, a total of three 10 μg/mL doxycycline-treated-group SCID mice and C3H mice, and a total of seven 50 μg/mL-doxycycline-treated-group SCID mice and C3H mice. A summary of the experimental design is shown in Figure 1.

#### BacLight Staining for Viability

To determine the proportion of live and dead *B. burgdorferi* following doxycycline treatment, Live/Dead BacLight^^®^^ (Molecular Probes) staining was performed on untreated and 50 μg/mL-treated *B. burgdorferi* per the manufacturer’s instructions. Briefly, 1.0 μL of 1.67 mM SYTO-9, a green nucleic acid stain, and 1.0 μL of 1.67 mM propidium iodide, which will only stain cells with damaged cell membranes red, were thawed and mixed in equal proportions. A volume of 1.5 μL of the stain mixture was then added to 500 μL of *B. burgdorferi* suspended in phosphate buffered saline, pH 7.4 (Gibco), incubated in the dark for 15 min, then applied to a slide and coverslip for viewing and counting. The samples were viewed and counted in live/dead ratios using a fluorescent microscope. The excitation/emission maxima for SYTO-9 is 480/500 nm, and 490/635 nm for propidium iodide. Images captured were obtained using a Nuance FX^^®^^36 fluorescence microscope (Leica) and software, with an optimal emission filter range of 500–560 nm for SYTO-9, and 600–650 nm for propidium iodide. Results were calculated as percent viability, and reported as mean ± SD per group.

#### RT-PCR

Mouse tissues collected from the assessment of infectivity by antibiotic-treated spirochetes were subjected to RT-PCR on the *flaB* and *ospC* genes as described (Embers et al., 2012). *B. burgdorferi* in these tissues was detected by a nested RT-PCR program utilizing 5S and 23S-targeted primers (Postic et al., 1994). This included external forward and reverse primers (CTGCGAGTTCGCGGGAGA-3′fwd; 5′-TCCTAGGCATTCACCATA-3′rev) and internal forward and reverse primers (5′-GAGTAGGTTATTGCCAGGGTTTTATT-3′fwd; 5′-TATTTTTATCTTCCATCTCTATTTTGCC-3′rev) targeting the 5S-23S intergenic sequence. Several genes identified as differentially expressed post-antibiotic treatment were subjected to standard RT-PCR to confirm the trend in gene expression. RNA was extracted from *in vitro*-cultured *B. burgdorferi* from each condition (untreated, treated and treated/re-grown) using the RNeasy kit.

A total of 10 ng RNA was added as template for the Qiagen^^®^^ One-step RT-PCR kit. Briefly, 0.4 nM of forward primer and 0.8 nM of the reverse primer were added to each reaction. The recommended cycling steps were used with annealing temperature up to 5°C below the melting temperature of each primer. Primers for amplification included: BB0778 = 5′TGTATGCACTGGTAGAAATA-3′(fwd) and 5′-TTTAACCTCTCCGTCTTTAT-3′(rev); BBA66 = 5′-GTTACAACCGTACCCGGAAATA-3′(fwd) and 5′-GCTGTCTTGGTTGACTAAAG-3′(rev); BBL39 = 5′-GGTGCTTGCAAGATTCATACTTC-3′(fwd) and 5′-CTCCTAAGTCTGCCCAGTTATT-3′(rev); BBN39 = 5′AGGAATCAGAAGACGAAGGATTAG-3′(fwd) and 5′-AACTTAGGCTCTTCGTAACCAG-3′(rev); BBJ09 = 5′-CAAGCTAAAGAGGCCGTAGAA-3′(fwd) and 5′-GCAAGCTTTGTATTGTTCGTAGT-3′(rev). Reactions were performed in a volume of 25 μl and 12 μl of each reaction was loaded onto the gel.

#### Preparation of RNA for Sequencing

*Borrelia burgdorfer*i cultures were grown to 5 × 10^7^ cells/mL for the control, treated, and treated/regrown groups in 50 mL conical tubes in BSK-II media. For the control group, total RNA was extracted, while the treated and treated/regrown groups were treated with 50 μg/mL doxycycline for 5 days. On day 5, total RNA from the treated *B. burgdorferi* culture was extracted, while the treated/regrown *B. burgdorferi* culture was resuspended in doxycycline-free media, and monitored for regrowth. When the culture regrew to the initial concentration, total RNA was extracted. RNA extraction was done according to the Qiagen^^®^^ RNEasy^^®^^ Mini Kit manufacturer’s instructions. In all cases, RNA was kept in a –80° freezer when not being processed, and freeze-thaw cycles were kept to a minimum. After RNA extraction, the total RNA was assessed for concentration and purity with a ThermoFisher Scientific^^®^^ NanoDrop^TM^ 2000 spectrophotometer. Samples with a total RNA concentration less than 300 ng/μL were not used in downstream steps. Next, for all groups, ribosomal RNA was depleted from the samples using Invitrogen Ribominus^^®^^ kits that followed the manufacturer’s instructions. Finally, the RNA for all groups was then processed according to the Life Technologies Ion Total RNA-Seq kit v2 sample preparation instructions. RNA integrity was verified using a BioAnalyzer (Applied Biosystems) prior to RNASeq.

#### Analysis of RNASeq Data

The *B. burgdorferi* genome FASTA and gene annotation GTF files were downloaded from PATRIC^1^ and the NCBI^2^ databases. After sequencing, the reference FASTA and annotation files were created from the *B. burgdorferi* chromosome and plasmids using a bash script and the sequenced samples were aligned using Bowtie2 (Langmead and Salzberg, 2012). Read counts were calculated for each gene using Htseq, and the software package DESeq2 was used to calculate differential gene expression between conditions (Anders et al., 2015). A cutoff of *p* < 0.05 using a Bonferroni False Discovery Rate (FDR) test, included in the DESeq2 package, was used to assess statistical significance between experimental conditions.

#### Construction of the http://borreliarna.tech Database

The database was constructed using a typical Drupal 7.x installation. A node was created for each gene, and using the pages module, html tables were created to display information for entire plasmids and the chromosome. Drupal indexed information for each node (webpage), which allowed the genes to be searchable using a search field. The data from the RNASeq experiment was also uploaded to the nodes, and designed to be searchable. The raw data are also available on the SRA (NCBI) database (accession # SUB5253665).

## Results

### Doxycycline Levels That Prevent Re-growth of *B. burgdorferi* in Culture Do Not Uniformly Prevent Infectivity of the Spirochetes in Mice

Three conditions of *B. burgdorferi* were tested for infectivity in mice (Figure 1) including untreated, 10 μg/mL doxycycline-treated, and 50 μg/mL doxycycline-treated. The doxycycline concentration of 10 μg/mL was utilized to serve as a treatment that is higher than the MIC but below the MBC, and the doxycycline concentration of 50 μg/mL was utilized based on previous work that determined it as the MBC for doxycycline (Caskey and Embers, 2015). The untreated control had 100% motile *B. burgdorferi* in the culture, the culture treated with 10 μg/mL doxycycline had approximately 20% motile *B. burgdorferi*, and the culture treated with 50 μg/mL had 0% motile *B. burgdorferi*. We hypothesized that doxycycline treatment would inhibit growth of the spirochetes, but not kill them. Thus, we surmised, they should grow in the *scid* mice, but not in the immunocompetent (C3H) mice because dormant or slow-growing spirochetes may not be able to evade the immune response of the host. As shown in Table 1, fewer mice were colonized (as indicated by organ culture) by the antibiotic-treated *B. burgdorferi*, but positive organ cultures were apparent from both C3H and *scid* mice. One single positive culture was used to score them as positive. In the initial experiment, the untreated *B. burgdorferi* were grown for 5 days after reaching a density of 5 × 10^7^ and had reached stationary phase prior to inoculation of mice. Thus, control mice (injected with 0 μg/mL-treated spirochetes) were not uniformly positive. For mice inoculated with 10 μg/mL-treated *B. burgdorferi* versus 0 μg/mL-treated *B. burgdorferi*, no significant differences in colonization were observed. The only somewhat significant difference was the number of *scid* mice infected with 50 μg/mL-treated *B. burgdorferi* versus 0 μg/mL-treated *B. burgdorferi* (Fisher’s exact test, 2-tailed *p* = 0.0885).

**Table 1:** Growth of antibiotic-treated *B. burgdorferi* in mice.

Treatment	Organ culture positive	RT-PCR positive
a. Results^∗^ with *scid* mice (3 combined experiments)
0 μg/mL	4/5	3/3
10 μg/mL	3/3	2/3
50 μg/mL	0/7	0/4
b. Results with C3H mice (2 separate experiments)
0 μg/mL	2/5 and 2/2	3/3 and 2/2
10 μg/mL	1/3 and 0/4	2/3 and 3/4
50 μg/mL	1/7 and 1/4	0/4 and 1/4

^∗^*number of mice positive/total mice tested*.

With respect to the molecular detection, mouse tissues were tested by RT-PCR for viability. Using several different targets, mice treated with 0 or 10 μg/mL were reliably positive, which correlated well with culture results. For those treated with 50 μg/mL of doxycycline, only one mouse was positive by RT-PCR and this result was found in the joint tissue by a nested set of primers. Amplification of *B. burgdorferi* genes from ear and heart tissue of mice inoculated with antibiotic-treated spirochetes is shown in Supplementary Figure 1.

### Genes That Are Upregulated Following Doxycycline Treatment May Reflect Entry Into Dormancy

**Figure 2:**
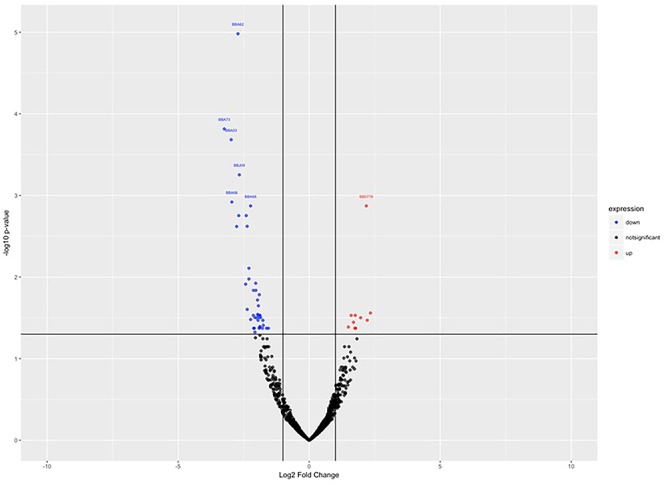
Volcano plot, showing the statistically significant gene expression changes in control (untreated) versus treated samples. Log2 fold change is plotted on the *x*-axis, and *p*-value (significance) on the *y*-axis, where the up-regulated genes are shown in red (right) and the down-regulated genes are shown in blue (left).

**Figure 3:**
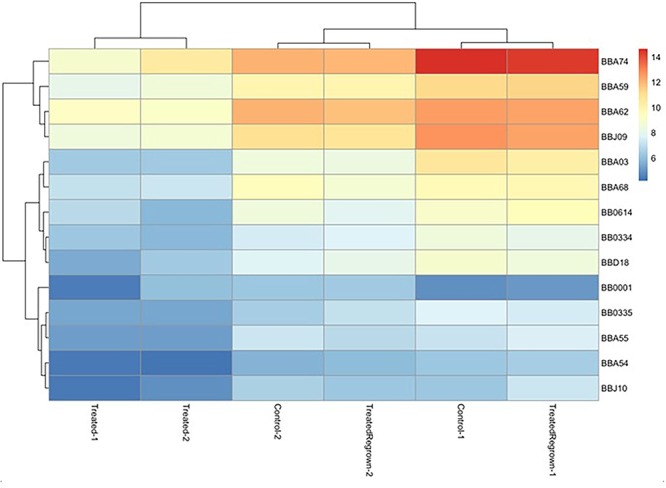
Heat map, indicating the most significantly different gene expression among groups. Relatedness among gene expression resulted in the pairing of treated group replicates together and untreated/treated regrown replicates together, where red is up-regulated and blue is down-regulated. Only the most significantly affected gene differences are shown, for simplicity.

**Figure 4:**
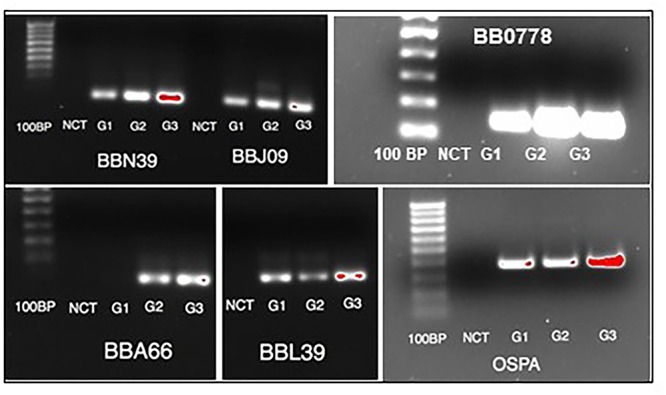
Validation of select gene expression by RT-PCR. RNA extracted from each of the three groups (G1 = untreated; G2 = treated; G3 = treated/regrown) was subjected to standard RT-PCR for significantly perturbed genes identified by RNASeq. NCT = negative control (no template).

To investigate the gene expression of *B. burgdorferi* during treatment with doxycycline and regrowth, three sample groups of *B. burgdorferi* were grown in BSK-II as untreated (G1), treated (G2), and treated/regrown (G3), where untreated was an untreated control, treated were spirochetes treated with the MBC of doxycycline, and regrown were spirochetes treated with the MBC of doxycycline then monitored for regrowth (see Figure 1, “Experimental Design”). Figure 2 shows a “volcano” plot comparing the significantly affected genes in doxycycline-treated versus untreated *B. burgdorferi.* Using the log2 fold change above 2, the number of significantly up-regulated genes was 20 and the number of down-regulated genes was 40. A heat map comparing the groups is shown in Figure 3. During treatment, the *bb0778* gene, which encodes the ribosomal protein L21, was significantly upregulated (*p* < 0.05; Ojaimi et al., 2003). This upregulation suggests that the doxycycline bound only to the 30S subunit, and not to other lower-affinity binding sites in the 50S ribosome, as has been proposed as a mechanism of action (Schnappinger and Hillen, 1996; Korobeinikova et al., 2012). Also, upregulated in the treatment versus control group were three Erp proteins (ErpA, ErpN, and ErpQ). The paralogous gene products of *bbP38* (ErpA) and *bbL39* (ErpN) were also subjected to RT-PCR to confirm the differential regulation (Figure 4). Operon-associated Erp proteins were also affected, but did not make the log fold change and significance cut-off in all cases. In order of upregulation in the treatment group were Erps A, N, P, G, B, and O. A compilation of the data showing relative fold changes and *p*-values can be found at the borreliarna.tech website. The expression of one gene, *bba66*, was increased in both the treated and the treated/regrown, whereas the others were also expressed, albeit at a lower level, in the untreated samples. The majority (50%) of the genes that were significantly differently expressed (*p* < 0.05) after treatment with doxycycline were found on lp54 (designated as *BBA*.._), most of which were down-regulated (Table 2). This includes outer surface protein-encoding genes associated with entry into the mammalian host (e.g., *osm28, p35, ospD, ospC, cspA*, and *dbpA*). The down-regulation of *ospA* was also observed; this may reflect a global down-regulation in gene expression and representative of the *in vitro* phenotype as *ospA* is down-regulated upon primary infection.

**Table 2:** Comparison of gene expression in treated *B. burgdorferi* versus. untreated.

*Gene ID*	Gene product description	Base mean	log2 fold change	lfcSE	Stat	*p*-value	*p*_adj_
**Down-regulated in treated versus control**				
*BB_I39*	Surface antigen	531.6302	-3.511814411	0.7	-5	0.000000459	0.000399019
*BB_A03*	Outer membrane protein	803.6569	-3.391446082	0.69	-5	0.000000803	0.000399019
*BB_A74*	Osm28 a periplasmic protein associated with the outer membrane	10746.24	-3.353348209	0.72	-5	0.00000296	0.000749933
*BB_J41*	Antigen P35	519.6167	-3.340637913	0.68	-5	0.00000108	0.000399019
*BB_A66*	Outer surface protein	506.0097	-3.084478479	0.75	-4	0.0000424	0.006202062
*BB_A73*	Antigen P35	244.0661	-3.070766419	0.73	-4	0.0000238	0.004300264
*BB_J09*	Outer surface protein D	3180.978	-3.006034687	0.65	-5	0.00000405	0.000854747
*BB_B19*	Outer-surface protein C (OspC)	1071.532	-2.845974147	0.77	-4	0.000203024	0.012794505
*BB_A62*	6.6 kDa outer membrane lipoprotein associated with tick phase and tick to mammal transmission	3657.207	-2.824080579	0.58	-5	0.00000126	0.000399019
*BB_A24*	dbpA; decorin-binding protein A	127.8711	-2.805940657	0.74	-4	0.000159342	0.010728237
*BB_A07*	ChpAI protein	43.45071	-2.733823725	0.81	-3	0.000734379	0.026765419
*BB_A36*	Lipoprotein	203.2405	-2.693016432	0.77	-3	0.000505215	0.022842927
*BB_A69*	Putative surface protein	765.4393	-2.65380562	0.7	-4	0.000136579	0.010728237
*BB_A55*	Hypothetical protein; pseudo	92.51022	-2.557310919	0.64	-4	0.0000588	0.006202062
*BB_0418*	*B. burgdorferi* predicted coding region BB0418	111.5509	-2.553114977	0.63	-4	0.0000495	0.006202062
*BB_D18*	Hypothetical protein	274.0594	-2.535299238	0.65	-4	0.0000935	0.00910675
*BB_I18*	Hypothetical protein	168.1778	-2.495501595	0.64	-4	0.00010117	0.00914866
*BB_0614*	Hypothetical protein	303.1408	-2.438966776	0.64	-4	0.000137231	0.010728237
*BB_A15*	Outer-surface protein A (OspA)	11478.47	-2.355995024	0.64	-4	0.000212231	0.012794505
*BB_A68*	cspA; complement regulator-acquiring surface protein 1	604.6092	-2.349991212	0.58	-4	0.000055	0.006202062
*BB_A51*	Hypothetical protein	31.06086	-2.316886337	0.75	-3	0.0018946	0.04502573
*BB_A16*	Outer surface protein B (OspB)	10183.59	-2.227444559	0.69	-3	0.001179972	0.035567733
*BB_J10*	Pseudo	42.55714	-2.155197984	0.69	-3	0.001874459	0.04502573
*BB_A41*	Hypothetical protein	124.9517	-2.149796432	0.61	-4	0.000445432	0.021831089
*BB_A46*	Hypothetical protein	105.1091	-2.095954433	0.63	-3	0.000890832	0.030480901
*BB_A76*	FAD-dependent thymidylate synthase (thyX)	464.0081	-2.095950278	0.62	-3	0.000761102	0.026765419
*BB_A59*	Hypothetical protein	1454.742	-2.090827439	0.63	-3	0.000966486	0.032199246
*BB_A50*	Pseudo	76.41753	-2.088161585	0.67	-3	0.001920529	0.04502573
*BB_I36*	Antigen P35	52.75825	-2.084527737	0.68	-3	0.002044638	0.047063839
*BB_0153*	Superoxide dismutase	619.057	-2.083171994	0.62	-3	0.000748591	0.026765419
*BB_A40*	Hypothetical protein	164.0255	-2.081525231	0.61	-3	0.000647805	0.026619772
*BB_0228*	Hypothetical protein	459.932	-2.079281862	0.67	-3	0.001846922	0.04502573
*BB_I29*	Virulence associated lipoprotein	61.1712	-2.077972331	0.66	-3	0.001663928	0.043886096
*BB_0334*	Peptide ABC transporter ATP-binding protein (OppD)	209.2004	-2.074544721	0.64	-3	0.001175603	0.035567733
*BB_0539*	Hypothetical protein	610.2785	-2.050851997	0.67	-3	0.002191659	0.048508182
*BB_0839*	Hypothetical protein	65.4991	-2.049111687	0.65	-3	0.001751913	0.044454875
*BB_0417*	Adenylate kinase	112.5984	-2.048758137	0.62	-3	0.000994892	0.032295719
*BB_0335*	Peptide ABC transporter ATP-binding protein (OppF)	143.0424	-2.046159104	0.63	-3	0.001064589	0.033694243
*BB_0843*	Arginine-ornithine antiporter	351.7582	-1.950882657	0.64	-3	0.002298966	0.048508182
*BB_0729*	Dicarboxylate/amino acid:cation symporter	214.2514	-1.912156352	0.62	-3	0.002113701	0.04778474
***Up-regulated in treated versus control***				
*BB_N39*	Protein ErpP	240.4476	1.803026693	0.59	3.1	0.002274471	0.048508182
*BB_P22*	Hypothetical protein	131.8377	2.063417184	0.66	3.1	0.001634559	0.043886096
*BB_L39*	ErpA8 protein (ErpN)	530.2168	2.073914497	0.61	3.4	0.000672854	0.026619772
*BB_P30*	Hypothetical protein	818.8261	2.111903193	0.63	3.4	0.000747768	0.026765419
*BB_P38*	Protein ErpA	509.9553	2.161061922	0.61	3.5	0.000398916	0.021831089
*BB_S01*	Phage portal protein	284.267	2.167560004	0.71	3.1	0.002275663	0.048508182
*BB_S22*	Pseudo	137.1996	2.177302565	0.68	3.2	0.001382682	0.039783546
*BB_L22*	Hypothetical protein	118.8342	2.200131032	0.69	3.2	0.001459404	0.041057898
*BB_P02*	Hypothetical protein	116.8938	2.275787679	0.67	3.4	0.000661851	0.026619772
*BB_0778*	rplU; 50S ribosomal protein L21	3695.16	2.279022028	0.56	4.1	0.0000476	0.006202062
*BB_P40*	Hypothetical protein	111.9668	2.312848106	0.74	3.1	0.001755722	0.044454875
*BB_N42*	Hypothetical protein	46.6144	2.347766937	0.74	3.2	0.001498845	0.041250811
*BB_P42*	Phage terminase large subunit	96.39695	2.364301812	0.73	3.2	0.001259505	0.037082184
*BB_M01*	Phage portal protein	267.0207	2.370596895	0.67	3.5	0.000424917	0.021831089
*BB_L01*	Phage portal protein	316.6145	2.401784081	0.69	3.5	0.000525561	0.022943435
*BB_P01*	Phage portal protein	337.2311	2.44942841	0.7	3.5	0.000448348	0.021831089
*BB_L41*	Hypothetical protein	102.397	2.486543676	0.71	3.5	0.000485634	0.022770844
*BB_L42*	Hypothetical protein	98.17613	2.640750924	0.73	3.6	0.00030885	0.017772912
*BB_P41*	Hypothetical protein	112.8184	2.652744102	0.7	3.8	0.000156214	0.010728237
*BB_L43*	Phage terminase large subunit	94.29416	2.720267979	0.72	3.8	0.000161008	0.010728237

As expected, a large number of genes were down-regulated in the treated group versus the untreated group. These are associated with positive growth regulation, infection/virulence and metabolism. These included virulence factors, peptide and nucleic acid transporters, superoxide dismutase, and adenylate kinase.

### Genes That Are Up-Regulated by *B. burgdorferi* Following Treatment and Re-growth May Be Associated With Exit From Dormancy

Many of the genes that were down-regulated upon treatment, were subsequently, and not surprisingly, up-regulated during re-growth (Table 3). Those genes that are upregulated in treated/regrown *B. burgdorferi* compared to those untreated may be of primary importance in establishing re-growth. In particular, *bba03, bba74 (oms28), ospA, ospD, bba62, cspA* and the oligopeptide permease protein genes *oppF* and *oppD* were up-regulated. Several of these gene products (outlined in the discussion) are associated with the habitation within the tick, and may therefore be indicators of dormancy, or exit from dormancy when expressed upon the blood meal (Caimano et al., 2016).

**Table 3:** Comparison of gene expression in treated and regrown *B. burgdorferi* versus treated only.

Gene ID	Gene product description	Base mean	log2 fold change	lfcSE	Stat	*p*-value	*p*_adj_
**Up-regulated in regrown *versus* treated**				
*BB_A03*	Outer membrane protein	572.4995	2.883544	0.5903	-4.88488	1.03E-06	0.000923
*BB_A74*	Osm28 a periplasmic protein associated with the outer membrane	8531.673	2.880761	0.625713	-4.60397	4.15E-06	0.001071
*BB_I39*	Surface antigen	286.618	2.734333	0.58228	-4.69591	2.65E-06	0.000923
*BB_J09*	Outer surface protein D	2587.885	2.638443	0.579187	-4.55542	5.23E-06	0.001071
*BB_J41*	Antigen P35	293.9898	2.607348	0.576747	-4.52079	6.16E-06	0.001071
*BB_J10*	Pseudo	65.80785	2.527191	0.618168	-4.08819	4.35E-05	0.006478
*BB_A62*	6.6 kDa outer membrane lipoprotein associated with tick phase and tick to mammal transmission	3028.1	2.505728	0.53339	-4.69774	2.63E-06	0.000923
*BB_A55*	Hypothetical protein; pseudo	91.59374	2.364567	0.596799	-3.96208	7.43E-05	0.00861
*BB_0614*	Hypothetical protein	321.2092	2.284102	0.613485	-3.72316	0.000197	0.018655
*BB_D18*	Hypothetical protein	223.7287	2.249952	0.559689	-4.02	5.82E-05	0.007588
*BB_A69*	Putative surface protein	568.7318	2.18589	0.60856	-3.59191	0.000328	0.026337
*BB_A68*	cspA; complement regulator-acquiring surface protein 1	521.7471	2.077714	0.548417	-3.78856	0.000152	0.015804
*BB_A59*	Hypothetical protein	1452.77	2.056081	0.566537	-3.62921	0.000284	0.02471
*BB_A15*	Outer-surface protein A (OspA)	10027.5	2.016905	0.60385	-3.34008	0.000838	0.054598
*BB_0839*	Hypothetical protein	66.6184	2.004598	0.587771	-3.41051	0.000648	0.045087
*BB_0335*	Peptide ABC transporter ATP-binding protein (OppF)	133.4096	1.94966	0.550044	-3.54455	0.000393	0.029299
*BB_0334*	Peptide ABC transporter ATP-binding protein (OppD)	174.8033	1.828725	0.554848	-3.2959	0.000981	0.060191
***Down-regulated in Regrown versus Treated***				
*BB_P42*	Phage terminase large subunit	90.95504	-2.00516	0.637717	3.144283	0.001665	0.096474

Several of these genes (*bbl39*/ErpN, *ospA*, *bbn39*/ErpQ) were much more abundant in the treated/re-grown compared to the untreated, indicating that they are associated with exiting dormancy rather than logarithmic growth, given that the growth phase was similar in the two groups.

## Discussion

The results of the bioassay in mice demonstrate that after treatment with the MBC of doxycycline, the spirochetes were able to host adapt, and evade immune pressure to establish an infection. The number of spirochetes injected into each mouse was held constant, but viability was not assessed. Therefore, the number of actively growing *B. burgdorferi* was likely significantly reduced after doxycycline treatment. What is most interesting about the result is that no significant differences were observed in the spirochete infectivity when immunocompetent versus immunodeficient mice were used. This result suggests that exit from dormancy may occur very rapidly *in vivo*, allowing the immunoevasive phenotype to become established.

We have previously demonstrated that the persister cell phenotype appears to be generated stochastically and driven by slowed growth (Caskey and Embers, 2015). In this study, we aimed to identify gene expression patterns associated with the survival and re-growth of *B. burgdorferi* in the antibiotic environment. While a similar study in which RNASeq was applied to antibiotic treatment of *B. burgdorferi* was conducted (Feng et al., 2015), several important distinctions between that study and ours should be made. In that report by Feng et al, *in vitro*-cultured Bb were treated with either doxycycline or amoxicillin (50 μg/mL) for 6 days and then subjected to RNASeq, with comparison to the untreated control. Genes that were up-regulated by 2-fold or more were ascribed significant and the pathways affected were elucidated. In our study, we treated a slightly denser culture (5 × 10^7^ versus 1 × 10^7^) with doxycycline at the same dose (50 μg/ml) for 5 days. An aliquot of the treated cells was allowed to re-grow, such that we had 3 treatment groups. In addition, we performed RNASeq on duplicate samples and ascribed significance using both fold-change and *p*-values to account for variation between samples. Both studies shed light on the mechanisms that Bb may use to establish persistence and re-grow. The Feng study identified a large number of genes as upregulated following treatment, whereas we found that the vast majority of genes were down-regulated, likely owing to a global decline in transcription. In the Feng study, the *ClpP* protease was indicated as the most highly up-regulated gene in treated *B. burgdorferi.* It was up-regulated in our screen as well (0.43-fold), but not determined to be significant. The genes found to be significantly increased in treated versus control encoded several Erp proteins and a 50S ribosomal protein (*bb0778*). This was verified by standard RT-PCR, as shown in Figure 4. The results in Figure 4 are derived from amplifying transcript from an equal quantity of input RNA. We did not have a good constitutively transcribed gene that is consistently expressed in all groups equally to be used as a housekeeping gene control, based on the RNASeq data. Thus, these results are not fully quantitative but only meant for validation of the RNASeq results.

We fully expected that genes categorized by involvement in the stress response (Bugrysheva et al., 2003; Drecktrah et al., 2015; Cabello et al., 2017) and DNA repair mechanisms (Fisher et al., 2017) would be significantly up-regulated with antibiotic treatment. However, this did not appear to be the case with either the Feng study or our own. The mechanisms governing the development of persister cells are not easily discerned by these RNASeq analyses, perhaps because the spirochetes stochastically enter dormancy (Caskey and Embers, 2015) and regrowth is determined by non-heritable traits. One supposition may be that instead, post-transcriptional, or even post-translational events such as lysine acetylation (Fisher et al., 2017; Bontemps-Gallo et al., 2018) govern the entry and exit from dormancy in the antibiotic environment. For example, in the toxin-mediated growth reduction within *S. typhimurium*, the toxins (TacT) add a post-translational modification (acetylation) to tRNA, which is reversed by a peptidyl tRNA hydrolase (Cheverton et al., 2016). A toxin-antitoxin system for *Borrelia* persister development has not been identified, but enzymatic modification of translational components could be involved in formation of persister cells and their re-growth. Nonetheless, we have identified a number of surface proteins which may be antigenic and serve as targets for novel immunotherapeutic strategies.

BBA62 is a 169 aa outer membrane protein located on plasmid lp54 that is annotated for North American and European strains of *B. burgdorferi.* It was found to be the most highly upregulated outer membrane protein in the treated/regrown group. BBA74 (Oms28), originally thought to be a porin (Skare et al., 1996), is an outer surface protein of 257 amino acids. The *bba74* gene is also located on plasmid lp54 it is thought to be expressed only in the tick and during blood-feeding, as it is induced by temperature shift within the tick, but not with host adaptation in the mammal (Mulay et al., 2007, 2009). That we are seeing it induced after doxycycline treatment is of interest and may parallel what we see with OspA (below). OspD is an outer membrane protein, located on plasmid lp38, which is known to be antigenic (Li et al., 2013) and expressed more highly in ticks than in the mammalian host (Li et al., 2007). BBA62 was described in 1997 as a 6.6 kD lipoprotein that did not appear to induce antibodies by animals needle-inoculated with *B. burgdorferi* (Lahdenne et al., 1997). The authors postulated that high-level expression of lp6.6 is associated with the arthropod phase of the spirochetal life cycle and that expression of the gene is downregulated during mammalian infection. OspA is known to be expressed in ticks and was the target of the only Lyme vaccine, which was shown to block transmission of *B. burgdorferi* (de Silva et al., 1996).

Analyses of *bba74* expression by primer extension of wild-type *B. burgdorferi* grown *in vitro*, along with *in vivo*-cultivated wild-type and *rpoS* mutant spirochetes, revealed that, like *ospA, bba74* is transcribed by sigma (70) and is subject to RpoS-mediated repression within the mammalian host. Meanwhile, *ospA, ospB*, and *ospD* appear to be regulated by the stress-response regulator *bosR* (Wang et al., 2013). These gene products are possible markers of dormancy, given their expression in ticks and in late Lyme arthritis (Li et al., 2013). In our study, *bosR* was shown to be up-regulated 0.325-fold following treatment and rpoS was up 0.386-fold; however, the *p*-values indicate that these were not significant perturbations. Interestingly, nine Erp proteins were found to be up-regulated by BosR (Ouyang et al., 2009), so perhaps even the slight increase in expression of this gene in response to doxycycline can have significant effects on gene regulation.

The use of RNASeq to study adaptation (Drecktrah et al., 2015; Arnold et al., 2016, 2018) has led to significant understanding of the changes in gene expression associated with growth phase and illumination of regulatory pathways. In this report, we add to this understanding through the analysis of the response to antibiotic, and elucidate possible antigenic targets (Oms28, OspA and several Erp proteins) for improved therapeutic intervention. The extension of these studies should be aimed at a better understanding of molecular adaptation to antimicrobial treatment *in vivo*.

## Ethics Statement

Practices in the housing and care of animals conformed to the regulations and standards of the PHS Policy on Humane Care and Use of Laboratory Animals, and the Guide for the Care and Use of Laboratory Animals. The Tulane National Primate Research Center is fully accredited by the Association for the Assessment and Accreditation of Laboratory Animal Care-International. The Institutional Animal Care and Use Committee of the Tulane National Primate Research Center approved all animal-related protocols, including the infection, treatment, and sample collection from mice.

## Author Contributions

JC performed research experiments and data analysis, and contributed significantly to writing. ME conceived the experiments, participated in analyses, and contributed significantly to writing. NH, DM, and MC performed the experiments and proofed the manuscript. VC contributed to the experiments and data analysis. RS contributed to the data analysis.

## Conflict of Interest Statement

The authors declare that the research was conducted in the absence of any commercial or financial relationships that could be construed as a potential conflict of interest.

## Footnotes

1 https://www.patricbrc.org/

2 https://www.ncbi.nlm.nih.gov/genbank/
