# Supplementary figures and images for: The Functional and Molecular Effects of Doxycycline Treatment on Borrelia burgdorferi Phenotype
Source: Front Microbiol. 2019 Apr 18;10:690. doi: 10.3389/fmicb.2019.00690 (PMC6482230; doi:10.3389/fmicb.2019.00690)

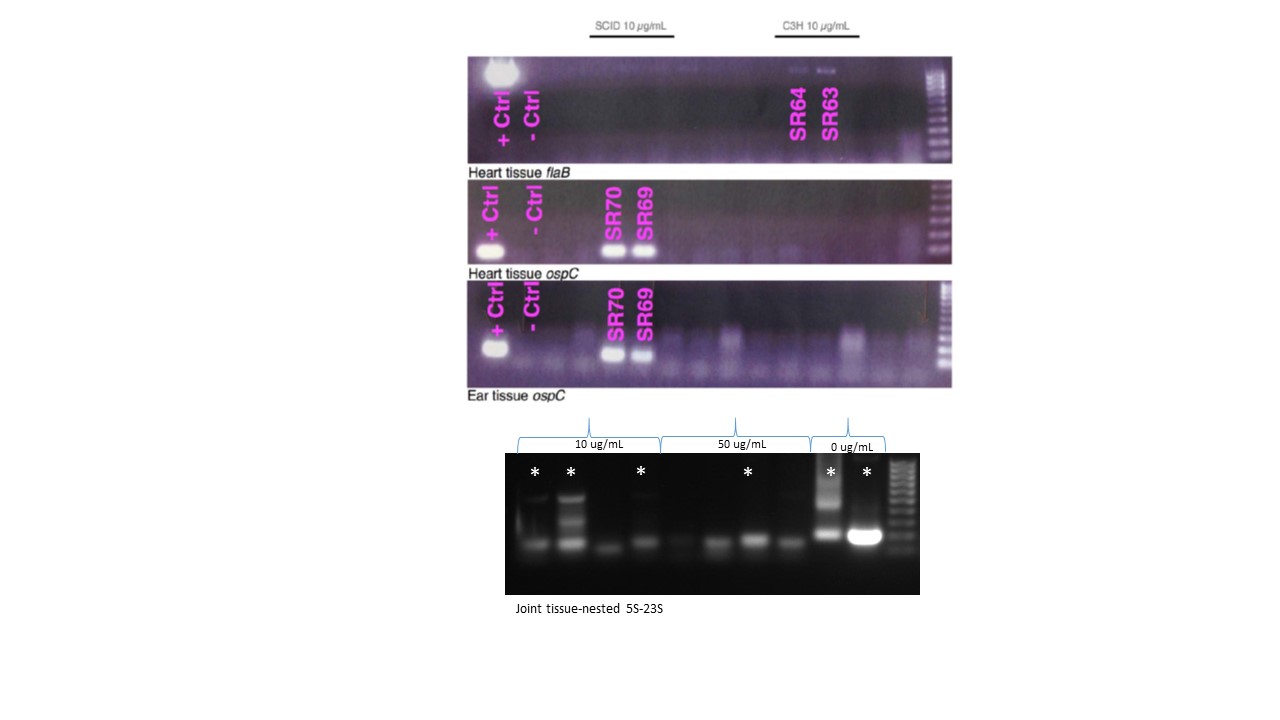

Supplement: Supplementary file 1 [file Image_1.JPEG]
