# Supplementary material for: The Functional and Molecular Effects of Doxycycline Treatment on Borrelia burgdorferi Phenotype
Source: Front Microbiol. 2019 Apr 18;10:690. doi: 10.3389/fmicb.2019.00690 (PMC6482230; doi:10.3389/fmicb.2019.00690)
Supplement: Supplementary file 2 [file Image_2.pdf]

BacLight Staining of Untreated and Treated *B. bugrdorferi*			
	Live	Dead	%Live
Control	155 +/- 18	12 +/- 9	92.8%
Treated (50 µg/mL)	21 +/- 4	37 +/- 15	36.2 %
Significance	$p < 2.2 \times 10^{-16}$, Fisher's Exact Test		

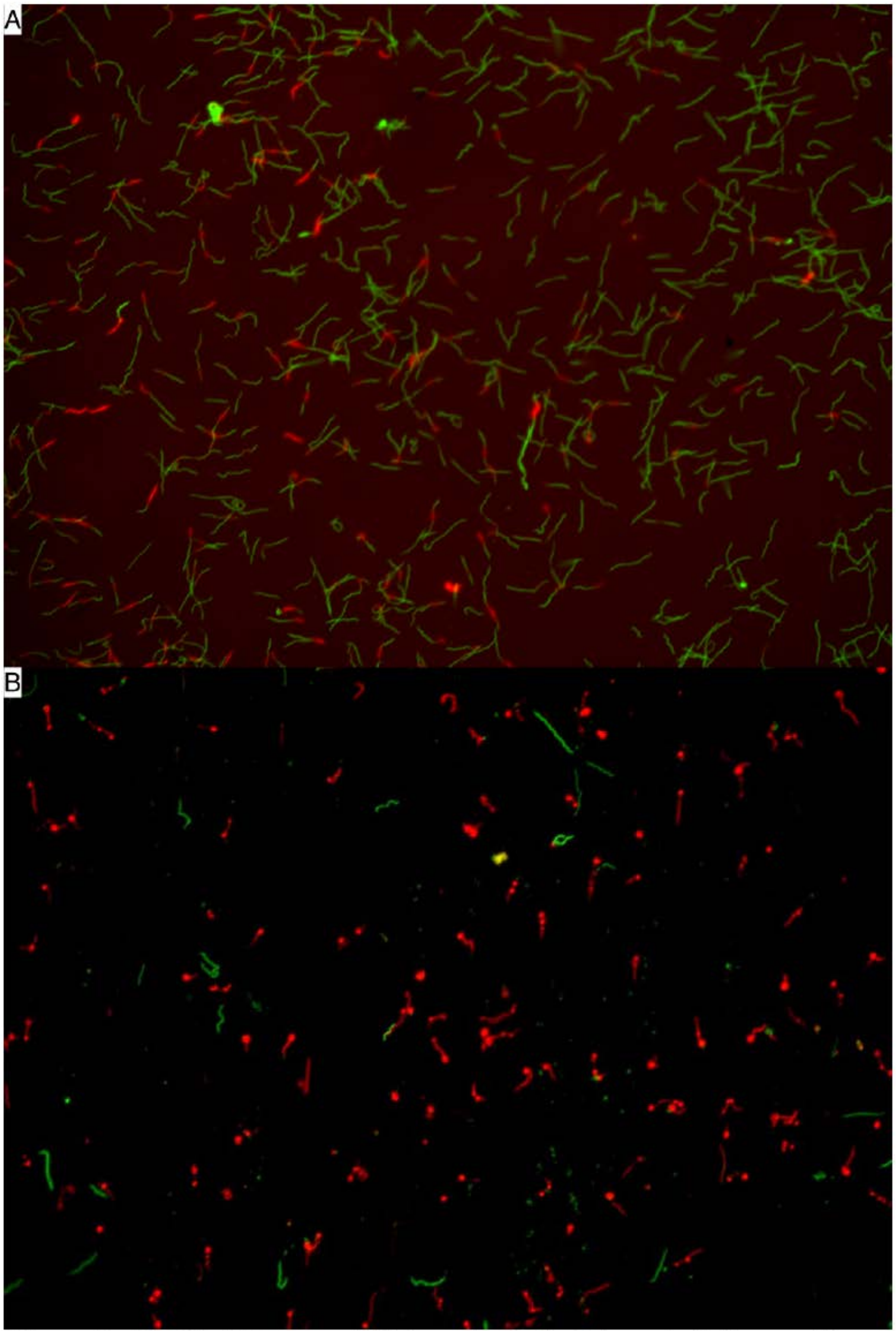

Supplemental Figure 2. BacLight images of *B. burgdorferi.* The spirochetes were either (A) not treated with antibiotic, or (B) treated with 50 µg/mL of doxycycline for 5 days. On day 5, the cultures were stained with the BacLight staining kit and observed by fluorescence microscopy.
